# Enhancement of CeO_2_ modified commercial SCR catalyst for synergistic mercury removal from coal combustion flue gas

**DOI:** 10.1039/d0ra04350h

**Published:** 2020-07-03

**Authors:** Shibo Zhang, Qingzhu Zhang, Yongchun Zhao, Jianping Yang, Yang Xu, Junying Zhang

**Affiliations:** Environment Research Institute, Shandong University Qingdao 266237 China zqz@sdu.edu.cn; State Key Laboratory of Coal Combustion, School of Energy and Power Engineering, Huazhong University of Science and Technology Wuhan 430074 China jyzhang@hust.edu.cn; School of Energy Science and Engineering, Central South University Changsha 410083 China

## Abstract

CeO_2_ modified commercial SCR (selective catalytic reduction) catalysts with different CeO_2_ content were prepared and researched for synergistic mercury removal from coal combustion flue gas in this study. The characterization analyses on the catalysts indicated that the introduction of CeO_2_ increased the surface area, the dispersity of the metal oxides on the TiO_2_ support and the redox behavior of the catalyst, which was beneficial to the catalytic activity. The experimental results confirmed that the CeO_2_ loading improved the catalytic efficiencies over the commercial SCR catalyst. The catalyst with a CeO_2_ content of 4% displayed the optimal performance for NO and synergistic Hg^0^ removal, of which the NO conversion and Hg^0^ removal efficiency reached 90.5% and 78.2%, respectively, at 300 °C in simulated coal-fired flue gas. The Hg^0^ removal activity, the independence of Hg^0^ removal from HCl concentration and the effects of SO_2_, NO and NH_3_ on Hg^0^ removal efficiency all became positive over the modified catalyst compared to over the raw one, which was mainly due to the sufficient chemisorbed oxygen derived from the synergy of V_2_O_5_ and CeO_2_ and the redox transformation between Ce^3+^ and Ce^4+^ on the catalyst surface. The CeO_2_ modification generated a significant enhancement on the catalytic performance and made the commercial SCR catalyst more suitable to be employed for NO and synergistic mercury removal in a coal combustion power plant.

## Abstract 2

CeO_2_ modification improves the synergistic Hg^0^ removal performance of commercial SCR catalyst.

## Graphical abstract

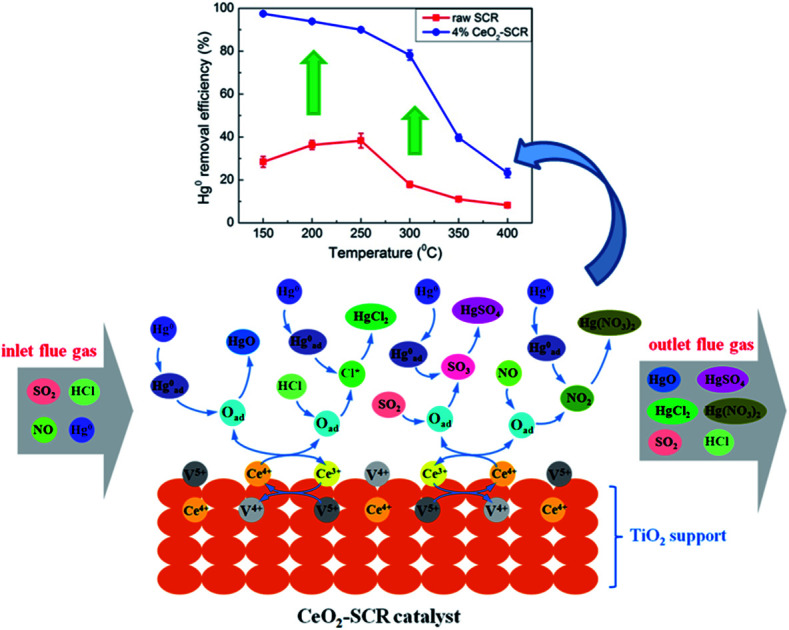

## 1. Introduction

Mercury is a kind of extremely harmful pollutant in the ecological environment. It poses a serious threat to human health due to its hypertoxicity, persistence and bioaccumulation.^1^ According to the Global Mercury Assessment 2018 issued by the UN Environment Programme, the global anthropogenic mercury emission reached 2150 tons in 2015, which increased by 12% compared to that in 2010.^2^ Significant coal burning is one of the main reasons for the growth of mercury emissions. And coal combustion power plants are considered as the major anthropogenic source of mercury release.^3^ As the Minamata Convention on Mercury came into force in August 2017, the limit on mercury emission from coal-fired power plants will be more rigorous on the basis of the existing regulations.^4^ Therefore, it is urgent to pay extensive attention to mercury emission control of coal combustion power plants under the dual pressure of environmental protection and convention fulfillment.

Mercury in coal-fired flue gas exists mainly in the types of elemental Hg (Hg^0^), oxidized Hg (Hg^2+^) and particle bound Hg (Hg^P^). Hg^2+^ and Hg^p^ can be respectively captured by wet flue gas desulfurization (WFGD) and particulate matter control device (PMCD) of power plant because of their physical properties, while Hg^0^ is difficult to be controlled by the single pollutant control equipment due to its volatility and water insolubility.^5,6^ So the key to the control of mercury emission from coal combustion power plant is the removal of Hg^0^. Similarly with mercury, NO_*x*_ is also a sort of hazardous contaminant with great harm to environment that coal burning releases, and NO occupies about 95% among NO_*x*_.^7–9^ Currently, the method of selective catalytic reduction (SCR) is generally used by coal-fired power plants for NO removal. Besides, the SCR catalyst has the capacity of oxidizing Hg^0^ to Hg^2+^ due to the existence of active oxygen on its surface, followed by Hg^2+^ being removed in the downstream WFGD.^10,11^ Compared with other Hg removal plans such as sorbent injection, utilizing SCR catalyst to synergistically remove Hg is remarkably cost-effective and meanwhile beneficial to the avoiding of secondary mercury pollution.^12^ Hence, it is promising for coal-fired power plant to adopt this approach to deal with the Hg removal from flue gas. And the research on the synergistic Hg^0^ oxidation with SCR catalyst has attracted more attention in recent years.

The commercial SCR catalyst that is currently used by coal combustion power plants is the TiO_2_-supported V_2_O_5_–WO_3_/TiO_2_ catalyst. A series of studies have been made on the Hg^0^ oxidation over the V_2_O_5_–WO_3_/TiO_2_ catalyst. The results indicated that the V=O bond on the catalyst surface could participate in Hg^0^ oxidation as the active sites. The Hg^0^ removal efficiency over the catalyst could reach 60–80% in general, and sometimes the efficiency was even higher than 90%.^13,14^ The increases of V_2_O_5_ loading, surface area and reaction temperature are in favor of the Hg^0^ oxidation activity.^15^ Especially, the existence of HCl in the flue gas had an obvious promotion on the Hg^0^ oxidation over the V_2_O_5_-based catalysts. Hg^0^ removal efficiency of V_2_O_5_–WO_3_/TiO_2_ was close to 100% at 380 °C with 4.5 mmol m^−3^ HCl contained in the reaction gas.^16^ The SiO_2_–TiO_2_–V_2_O_5_ catalyst likewise showed a Hg^0^ removal efficiency of nearly 100% in the co-presence of O_2_ and HCl.^17^ And the facilitation of HCl on the efficiency of commercial SCR catalyst was also testified by kinetic analysis.^18^ However, though the commercial V_2_O_5_–WO_3_/TiO_2_ catalyst displays certain Hg^0^ removal capacity under the appropriate conditions, it has apparent drawbacks such as the narrow working temperature range and the limited Hg^0^ removal efficiency at the SCR operating temperature.^16,19^ Meanwhile, the effectiveness of Hg^0^ removal depends heavily on the HCl concentration. The efficiency could be as high as 90% in the flue gas derived from burning high-rank coal, while in flue gas of burning low-rank coal only less than 30% was observed.^17,20,21^ This condition is distinctly disadvantageous to those power plants that combust sub-bituminous coal or lignite. So it is necessary to make modification on commercial SCR catalyst to improve its catalytic properties. In recent years, CeO_2_-based catalysts have gradually come into view of researchers due to its prominent catalytic activity. Related studies demonstrated that element Ce would help enhance the oxygen storage capacity of the catalyst, which led to the superior performance on NO and Hg^0^ removal. Illustratively, Gao *et al.*^22^ prepared CeO_2_/TiO_2_ catalyst by sol–gel method and found the NO conversion of the catalyst reached 93.4–98.6% in the wide temperature range of 250–450 °C; Li *et al.*^23^ investigated Hg^0^ removal activity of CeO_2_/TiO_2_ in simulated coal-fired flue gas and confirmed the optimal efficiency could attain 94%, and efficient Hg^0^ oxidation could be achieved even in the absence of HCl; Fan *et al.*^24^ acquired that the zeolite supported CeO_2_/HZSM-5 catalyst exhibited Hg^0^ removal efficiency of more than 95% among the range of 120–320 °C; Wang *et al.*^25^ loaded CeO_2_ on Ti-based pillared interlayered clays to examine the simultaneous NO and Hg^0^ removal efficiency over the catalyst, and the results showed that the NO conversion was almost 100% at 350 °C while Hg^0^ removal efficiency also reached higher than 50% in the same condition. In view of the advantage of the activity of catalyst containing CeO_2_, it is reasonable to speculate that using CeO_2_ to modify the V_2_O_5_–WO_3_/TiO_2_ catalyst will make a significant improvement on the catalytic properties of the catalyst. Zhao *et al.*^19^ has previously modified the TiO_2_ support with CeO_2_ and synthesized V_2_O_5_–WO_3_/TiO_2_–CeO_2_ catalyst, and the experimental study confirmed the enhancement of Hg^0^ removal performance of the catalyst, such as the efficiency and sulfur-resistance, resulted from the addition of CeO_2_. Some literatures also prepared the CeO_2_ modified V_2_O_5_–WO_3_(MoO_3_)/TiO_2_ to investigate the NO removal activity specifically, and the satisfactory NO conversions, sulfur-resistance and alkali metal resistance were obtained over the catalysts.^26–28^ Nevertheless, few literatures have made investigations on the effectiveness of employing CeO_2_ to directly modify the commercial SCR catalyst of power plant for synergistic Hg^0^ removal so far, which is of great value and close correlation to practical application. Moreover, the present commercial SCR catalyst is not replaceable in the short term, though some researched novel catalysts such as Mn-based, Cu-based, noble metal and perovskite structure catalysts displayed considerable Hg^0^ removal efficiency in the lab-scale tests.^29–32^ Thus, it can be seen that it is of great significance to examine the synergistic Hg^0^ removal performance of the CeO_2_ modified commercial V_2_O_5_–WO_3_/TiO_2_ catalyst.

Based on the above presentations, this work takes CeO_2_ modified commercial SCR catalyst as the researching object, and conducts the experiments in simulated coal combustion flue gas (SFG). NO removal performance of the catalysts with different CeO_2_ loadings were first tested considering the primary purpose of SCR. Then the Hg^0^ removal activity of the catalysts was investigated in detail. Hg^0^ removal efficiencies over different CeO_2_-loading catalysts at different temperatures were evaluated, and the effects of individual flue gas components in SFG on the efficiency were detected as well. The characterization analyses of X-ray fluorescence (XRF), Brunauer–Emmett–Teller (BET), X-ray diffraction (XRD), H_2_-Temperature Programmed Reduction (H_2_-TPR) and X-ray photoelectron spectroscopy (XPS) were carried out to understand physical–chemical properties of the catalysts and explore the modification mechanism of CeO_2_. The study results of this work will present application prospect of the CeO_2_ modification on commercial SCR catalyst for improving the catalytic performance.

## 2. Materials and methods

### 2.1. Catalyst preparation

The honeycomb commercial SCR catalyst employed in this study was got from a catalyst corporation of China which professionally produces SCR catalyst of coal-fired power plant. The CeO_2_ modified catalysts were prepared by the solution impregnation method. The honeycomb catalyst was grinded to powder first and sieved with a 200 mesh sifter. Then a certain amount of the sieved fine catalyst powder was placed in a beaker, followed by the Ce(NO_3_)_3_ aqueous solution which contained the desired quantity of Ce(NO_3_)_3_ being filled into the beaker. The obtained slurry was stirred for 1 h and then exposed to an ultrasonic bath for 2 h. After the mixture was dried at 110 °C for 12 h and calcinated in air at 500 °C for 4 h sequentially, the final CeO_2_ modified commercial SCR catalyst was acquired. The mass fractions of CeO_2_ of 1%, 2%, 4% and 7% in the modified catalysts were designed. In the process of preparing the catalysts with different CeO_2_ loadings, the weight of the original catalyst powder was remained unchanged, and the CeO_2_ loading was controlled by the solvend amount of the added Ce(NO_3_)_3_ aqueous solution. The CeO_2_ modified catalysts were abbreviated as (*x*)CeO_2_-SCR (*x* represents the mass fraction of CeO_2_) in the later sections, and the catalyst without modification was designated as raw SCR. Additionally, the pure CeO_2_ catalyst was also prepared for comparison, which used Ce(NO_3_)_3_ as the precursor as well to maintain the consistency.

### 2.2. Catalyst characterizations

The characterization methods of XRF, BET, XRD, H_2_-TPR and XPS were carried out over the fresh and spent catalyst samples in order to understand the physical and chemical properties of the catalysts and analyze the CeO_2_ modification mechanism. The XRF analysis was conducted with an EAGLE III focusing fluorescence spectrograph which was operated at 38 kV. The measurement of the BET surface was accomplished on an ASAP 2020 porosimeter by means of N_2_ adsorption. The XRD analysis was performed using an X'Pert PRO diffractometer (Cu Kα radiation) of which the working voltage and emission current were 40 kV and 40 mA, respectively, with the scanning angle ranging from 10° to 80° (2*θ*). The test of H_2_-TPR was carried out on an Autochem 2920 analyzer with the operating temperature raised from 30 °C to 850 °C at a rate of 10 °C min^−1^, and the reaction gas was 50 mL min^−1^ 10% H_2_/Ar. The XPS technique was implemented on a PerkinElmer PHI 5100 ESCA system with Al Kα X-ray source (*hν* = 1486.6 eV) to study the valence states of the elements and using the C 1s binding energy value of 284.6 eV for the spectra calibration.

### 2.3. Catalytic activity measurement

**Fig. 1:**
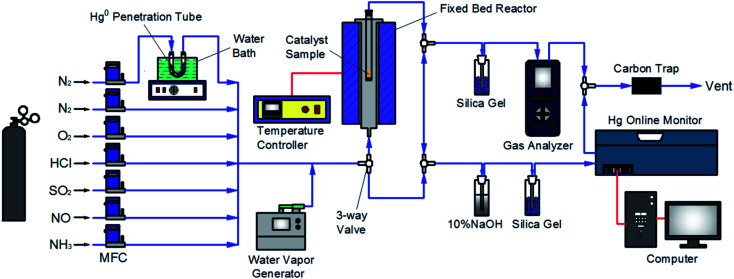
Schematic diagram of the experimental system.

The experimental system used in this work was similar to that employed in our previous studies,^33–35^ as described in Fig. 1. Briefly, the flue gas components (N_2_, O_2_, HCl, SO_2_, NO, and NH_3_) came from standard cylinder gases and their gas flow was accurately controlled by the corresponding calibrated mass flowmeter, respectively. Water vapor (H_2_O) was produced by a steam generator. The continuous feed of Hg^0^ vapor of approximately 60 μg m^−3^ was generated from a Hg^0^ penetration tube (VICI, Metronics Inc., Santa Clara, CA) which was placed in a U-tube and heated by a water bath, with N_2_ carrying the Hg^0^ vapor into the flue gas. The catalytic reaction was made to occur in a fixed bed reactor with a temperature controller to set the reaction temperature. The NO and Hg^0^ concentrations in the flue gas were measured by a gas analyzer (AFRISO, Multilyzer STe, M60) and a Hg^0^ online monitor (Ohio Lumex, RA-915M), respectively. And the N_2_O and NO_2_ concentrations were monitored with a FTIR analyzer (Gasmet Dx4000). Several specific gas-washing bottles were added for eliminating the acid gas to prevent corrosion and interferences on the monitoring devices. The gas line of the system was heated by electric heating belt to avoid any possible adsorption of the gas components on the line before the measurement. The exhaust gas was purified by active carbon before discharged to atmosphere.

The experiments of this work were carried out under the condition of simulated coal-fired flue gas of which the composition was 4% O_2_, 10 ppm HCl, 800 ppm SO_2_, 400 ppm NO, 400 ppm NH_3_, 8% H_2_O and 60 μg m^−3^ Hg^0^ with N_2_ to balance unless otherwise noted. The total flow of the flue gas was controlled at 1 L min^−1^. The catalyst dosage was 0.5 g for each test, and the space velocity (GHSV) was correspondingly about 50 000 h^−1^. In each test, the flue gas was first introduced to the bypass, and the concentrations of NO and Hg^0^ at the inlet of the reactor were acquired when the readings of the monitoring devices reached stability. Then the gas stream was switched to pass through the catalyst until the stable NO and Hg^0^ concentrations at the outlet of the reactor were obtained as well. The stability was defined as the fluctuation of the concentrations being no more than 5% for a period of at least 30 min. After each step of the experiment, the spent catalyst was replaced by fresh sample before starting the next test. The NO conversion, N_2_ selectivity and Hg^0^ removal efficiency adopted to evaluate the catalytic activity of the catalyst were respectively calculated by eqn (1)–(3) as follows.1
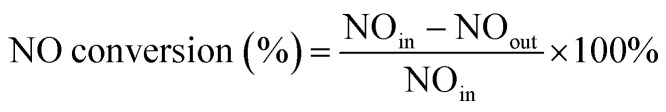
2
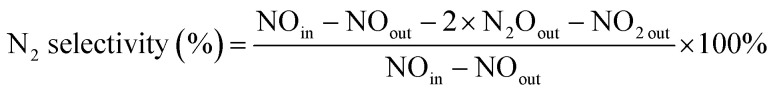
3
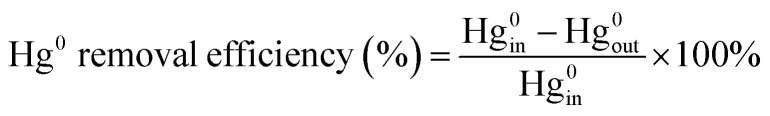


The subscript “in” and “out” in the equations represented the gas concentrations at the inlet and outlet of the reactor, respectively. As the outlet Hg^0^ concentration was read when it achieved a stable value, the catalyst was in the state of Hg saturated adsorption at this time and all the removed Hg was gaseous Hg^2+^. Additionally, the researched catalysts were verified to have almost no capacity for Hg^0^ removal at room temperature. So the physical adsorption of Hg^0^ was negligible, and the defined Hg^0^ removal efficiency here was equal to Hg^0^ oxidation efficiency.

## 3. Results and discussion

### 3.1. Characterization of the CeO_2_-SCR catalysts

#### 3.1.1 XRF analysis

XRF analysis was adopted to investigate the element compositions and contents of the catalysts. The results were summarized in Table 1. Before the loading of CeO_2_, the content of V_2_O_5_ which was the active component and the content of WO_3_ using for improving the thermal stability and surface acidity in raw SCR catalyst were 0.98% and 6.63%, respectively. Both the values were among the ranges of the contents of V_2_O_5_ and WO_3_ in usual honeycomb commercial SCR catalyst, which were respectively 0.5–3% and 2–10%. The activity of SCR catalyst was generally in proportion to the content of V_2_O_5_. But exorbitant vanadium content would lead to the growing SO_2_/SO_3_ conversion.^36^ The V_2_O_5_ content of the raw SCR catalyst employed in this work was a moderate percent of about 1%, indicating this catalyst was well typical and representative. Small amount of SiO_2_ was also detected to contain in the catalyst, which was helpful for boosting the mechanical strength. For the CeO_2_ modified catalysts, the practical contents of CeO_2_ in the catalysts with different CeO_2_ loadings were very close to the corresponding designed values, which testified the accuracy of the preparation of the catalysts. Meanwhile, the addition of CeO_2_ did not cause apparent variations on the contents of V_2_O_5_, WO_3_ and SiO_2_ in the catalysts.

**Table 1:** Element compositions and contents of the CeO_2_ modified commercial SCR catalysts

Catalyst	Mass fraction (%)				
	CeO_2_	TiO_2_	V_2_O_5_	WO_3_	SiO_2_
Raw SCR	0	90.71	0.98	6.63	1.68
1% CeO_2_-SCR	0.92	89.73	0.96	6.58	1.81
2% CeO_2_-SCR	1.95	88.54	1.08	6.80	1.63
4% CeO_2_-SCR	4.03	86.93	1.12	6.35	1.57
7% CeO_2_-SCR	6.79	84.18	1.20	6.06	1.77

#### 3.1.2 BET analysis

The surface structural properties of the CeO_2_ modified commercial SCR catalysts tested by BET analysis were listed in Table 2. According to the results, the surface area of the raw catalyst was at a relatively low level of 18.64 m^2^ g^−1^, which might result from the specific preparation process of the catalyst corporation. The introduction of CeO_2_ made a significant enhancement on the surface area and pore volume of the catalyst. The surface area increased dramatically from 18.64 m^2^ g^−1^ to 69.23 m^2^ g^−1^ with the loading of only 1% CeO_2_. The increase of surface area could raise the amount of the active sites available for Hg^0^ and other reactants on the catalyst surface, thereby it usually being beneficial to the catalytic activity.^35,37^ And the enlargement of pore volume was also in favor of the Hg^0^ removal capacity of the catalyst. The surface area showed a slight declined trend as the CeO_2_ loading augmented, which was probably due to the blockage of some surface micropores caused by the increasing CeO_2_ loading.^38,39^ It's worth noting that the surface area of the CeO_2_ modified catalysts was much closer to that of pure CeO_2_ than to the raw SCR catalyst, indicating that the surface area was obviously altered and controlled by CeO_2_ though it occupied only a minor proportion in the catalysts. By contrast, the pore size of the catalyst was not distinctly affected by the addition of CeO_2_, and the change was small.

**Table 2:** Surface structural properties of the CeO_2_ modified commercial SCR catalysts

Catalyst	BET surface area (m^2^ g^−1^)	Pore volume (cm^3^ g^−1^)	Pore size (nm)
Raw SCR	18.64	0.069	19.579
1% CeO_2_-SCR	69.23	0.287	16.555
2% CeO_2_-SCR	66.39	0.279	17.081
4% CeO_2_-SCR	64.92	0.285	17.248
7% CeO_2_-SCR	61.83	0.233	15.553
Pure CeO_2_	66.28	0.294	17.835

#### 3.1.3 XRD analysis

**Fig. 2:**
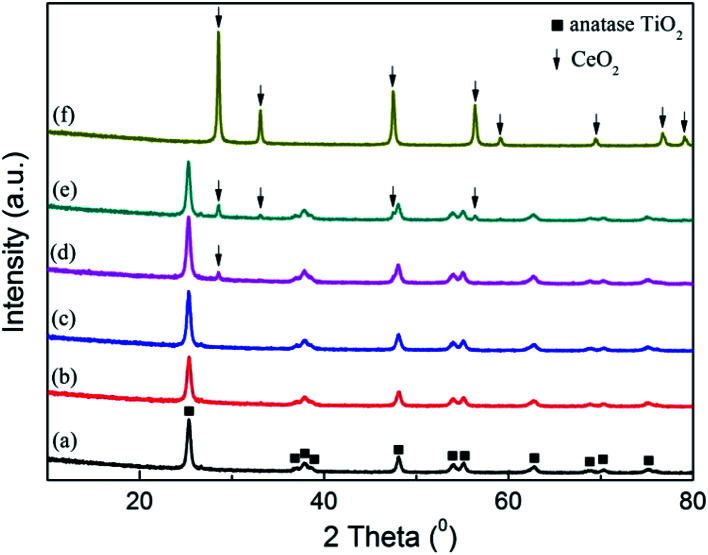
XRD patterns of the catalysts ((a) raw SCR, (b) 1% CeO_2_-SCR, (c) 2% CeO_2_-SCR, (d) 4% CeO_2_-SCR, (e) 7% CeO_2_-SCR, (f) pure CeO_2_).

The crystalline characteristic in the catalysts was investigated by XRD analysis, and the result was shown in Fig. 2. On the patterns of raw SCR catalyst and pure CeO_2_, only the peaks corresponding to anatase TiO_2_ and CeO_2_ were discovered respectively.^23,30,40^ With CeO_2_ doped into the commercial SCR catalyst, the peak intensity of TiO_2_ became weak gradually, and meanwhile the peak standing for CeO_2_ was not detected when the CeO_2_ content was lower than 4%. This phenomenon suggested that there existed some interaction between TiO_2_ and CeO_2_ in the catalysts.^33,41,42^ CeO_2_ was well dispersed and in the form of amorphous phase on the catalyst surface. As the CeO_2_ content reached 4%, a peak corresponding to CeO_2_ emerged on the pattern at 28.6°, indicating that the present load amount has made the dispersion of CeO_2_ on the catalyst reach the critical point of saturation. Further increasing the CeO_2_ loading would lead to the conversion of the doped CeO_2_ from amorphous phase to crystalline state. The emergence of distinct characteristic peaks corresponding to CeO_2_ on the profile of 7% CeO_2_-SCR confirmed this inference. In addition, the peaks of V_2_O_5_ and WO_3_ were not discovered on any catalyst pattern, displaying an amorphous distribution as well. More active substance existed in the amorphous phase was considered to be advantageous for the catalytic activity of the catalyst, while the appearance of the crystal of the active species was adverse to the catalytic performance.^43,44^

### 3.2. NO removal performance of the CeO_2_-SCR catalysts

**Fig. 3:**
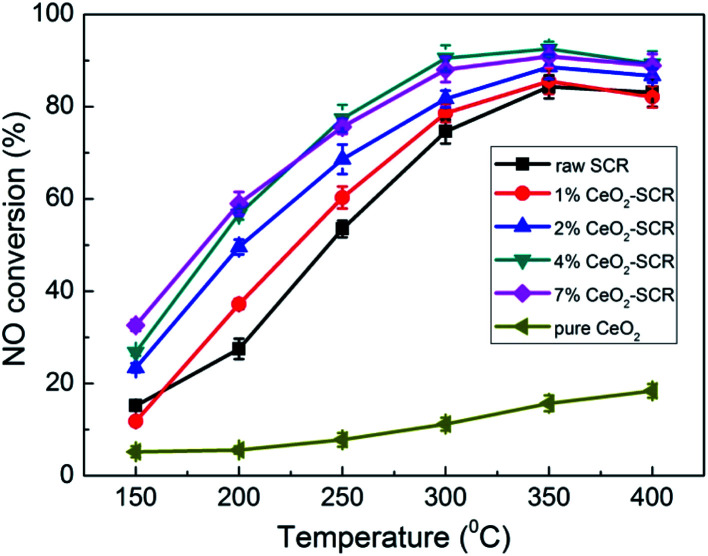
NO conversion over the CeO_2_ modified commercial SCR catalysts under different reaction temperatures in simulated coal-fired flue gas.

Considering the primary function of SCR catalyst was to remove NO for coal combustion power plant, NO removal activity of the CeO_2_ modified commercial SCR catalysts in simulated coal-fired flue gas was first examined prior to the investigation on Hg^0^ removal performance. The experimental results were shown in Fig. 3. The NO conversions of the catalysts showed a growing trend as the reaction temperature increased from 150 °C to 400 °C. The optimal temperature range was 300–400 °C which was consistent with that of literature report.^41,45,46^ NO conversion over the raw SCR catalyst in this range was 74.6–84.3%, which was a little lower than the efficiencies monitored in power plants. This might be attributed to the higher GHSV in the lab reactor than that under the practical conditions (2000–3000 h^−1^),^47^ which led to the shorter contact time between flue gas and catalyst. As CeO_2_ was added into the catalyst, NO conversion was apparently promoted. And the catalyst with the CeO_2_ loading of 4% exhibited the best activity for NO removal. The NO conversions were 90.5%, 92.5% and 89.3%, respectively, at the temperature points of 300–400 °C over 4% CeO_2_-SCR. Besides, the efficiency of 4% CeO_2_-SCR could also reach nearly 80% at 250 °C. Thus, the CeO_2_ modification not only improved NO conversion of commercial SCR catalyst, but also broadened the working temperature and enhanced the medium-low temperature activity of the catalyst. The superior NO removal performance of 4% CeO_2_-SCR was associated with the higher content of CeO_2_ dispersed in the amorphous phase, while the slightly decreased NO conversion over 7% CeO_2_-SCR compared to that over 4% CeO_2_-SCR might be due to the generation of CeO_2_ crystal in the catalyst. Additionally, the surface area was also a possible influence factor for the NO removal activity because the variation trend of the surface area was very close to that of the NO conversion among 4% CeO_2_-SCR, 7% CeO_2_-SCR and the raw catalyst. Therefore, the experimental acquirement was in good agreement with the characterization results. The efficiency of pure CeO_2_ was in a poor level among the testing temperature range, indicating that the element V was still responsible for the nice NO removal activity of CeO_2_-SCR though CeO_2_ generated modification effects on the catalysts. To sum up, the CeO_2_ modification led to an advancement upon the property of the commercial SCR catalyst and made it own prominent NO removal activity, which established a solid foundation on the utilization of the catalyst for synergistic Hg^0^ removal.

**Fig. 4:**
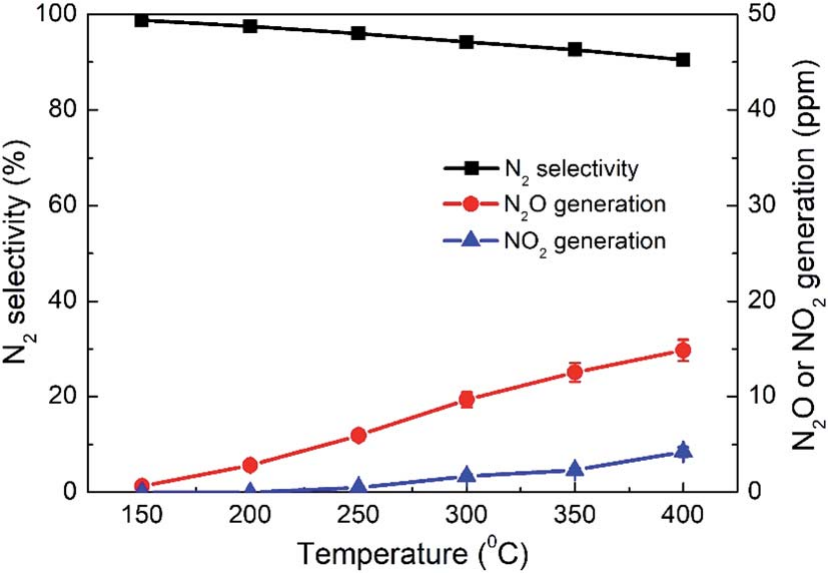
N_2_ selectivity and N_2_O and NO_2_ generations over 4% CeO_2_-SCR under different reaction temperatures in simulated coal-fired flue gas.

As another important evaluation index for NO removal performance, N_2_ selectivity was measured over the 4% CeO_2_-SCR catalyst which exhibited the highest NO conversion, and the results were shown in Fig. 4. Under SFG, the N_2_ selectivity over the catalyst reduced slightly with the increase of the reaction temperature, which was caused by the generation of a small amount of N_2_O and NO_2_ during the reaction. The detected concentrations of N_2_O were much higher than those of NO_2_. So the decrease of the N_2_ selectivity was mainly due to the N_2_O generation at the higher temperatures. Nevertheless, the N_2_O generation was lower than 15 ppm in the whole temperature range of 150–400 °C, and even the poorest N_2_ selectivity measured at 400 °C reached as high as 90.5%. Hence, the catalyst displayed great N_2_ selectivity in the NO removal process, further confirming the excellent NO removal performance of the CeO_2_-SCR catalyst in the simulated coal-fired flue gas.

### 3.3. Hg^0^ removal performance of the CeO_2_-SCR catalysts

#### 3.3.1 Hg^0^ removal efficiency under different temperatures in SFG

**Fig. 5:**
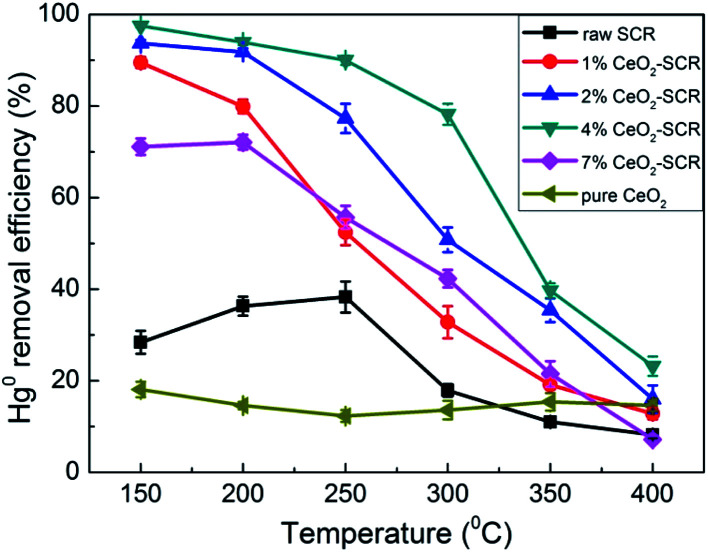
Hg^0^ removal efficiency over the CeO_2_ modified commercial SCR catalysts under different reaction temperatures in simulated coal-fired flue gas.

Hg^0^ removal performance of the CeO_2_ modified commercial SCR catalysts was then investigated as the emphasis. First, the Hg^0^ removal efficiencies of the catalysts in simulated coal-fired flue gas were measured under different reaction temperatures, and the results were shown in Fig. 5. As the temperature increased, the variation trend of the Hg^0^ removal efficiencies of the CeO_2_-SCR catalysts was opposite to that of the NO conversions, and it was a descending tendency. The possible reason for this phenomenon was that the lower temperature was beneficial to the Hg^0^ adsorption on the catalyst which was an essential procedure for Hg^0^ removal, and the Hg^0^ oxidation was realized mainly through the form of adsorbed Hg^0^ (Hg^0^_ad_).^35,42,48^ The introduction of CeO_2_ into the catalyst accelerated the Hg^0^ removal efficiency apparently. Analogously to the testing results of NO removal activity, the optimal sample for Hg^0^ removal was 4% CeO_2_-SCR as well, which corresponded to the characterization results again. Hg^0^ removal efficiency of 4% CeO_2_-SCR achieved more than 90% in the temperature range of 150–250 °C. Even at 300 °C which was among the conventional operating temperature of SCR catalyst (300–400 °C), 4% CeO_2_-SCR also exhibited the efficiency of as high as 78.2% on the basis of NO conversion guaranteed at 89.3%. So the catalyst showed remarkable activity for simultaneous NO and Hg^0^ removal. The prominent performance for synergistic Hg^0^ removal was mainly owed to the sufficient chemisorbed oxygen (O_ad_) of 4% CeO_2_-SCR led by the existence of Ce^3+^/Ce^4+^ ion pair and the oxygen transfer between them in the catalyst,^38,49^ which would be confirmed by the subsequent XPS analysis. The abundant O_ad_ would facilitate Hg^0^ oxidation to generate HgO as the active species. The related reaction process was described by eqn (4)–(6). As the efficiencies of the raw catalyst and pure CeO_2_ were no more than 38.3%, the superior performance of the CeO_2_ modified commercial SCR catalyst was also primarily resulted from the synergy of V_2_O_5_ and CeO_2_ in the catalyst.^50^ In addition, considering the GHSV was much higher in the experimental condition than in actual flue gas of power plant, the catalytic efficiencies might be preferable in practical application. Hence, the SCR catalyst manifested to be more competent and promising for commercial use after the CeO_2_ modification.

4 Hg^0^ (g) → Hg^0^_ad_

5 2CeO_2_ → Ce_2_O_3_ + O_ad_

6 Hg^0^_ad_ + O_ad_ → HgO

#### 3.3.2 Effects of the flue gas components on Hg^0^ removal efficiency

Effect of each flue gas component on the Hg^0^ removal efficiency of the CeO_2_-SCR catalyst was then investigated to reveal its role in Hg^0^ removal process. And the results were made comparison with those of the raw SCR catalyst to explore the reasons for the modification effect of CeO_2_ on the catalyst for Hg^0^ removal in simulated coal-fired flue gas. Because the optimum catalytic efficiencies were implemented at 300 °C over 4% CeO_2_-SCR with the NO conversion and synergistic Hg^0^ removal efficiency being 89.3% and 78.2%, respectively, the experiments of this part were carried out at 300 °C using 4% CeO_2_-SCR as the catalyst sample. The reaction atmosphere was SFG with the concentration of the investigated component changed and the others constant.

##### 3.3.2.1. Effect of HCl

**Fig. 6:**
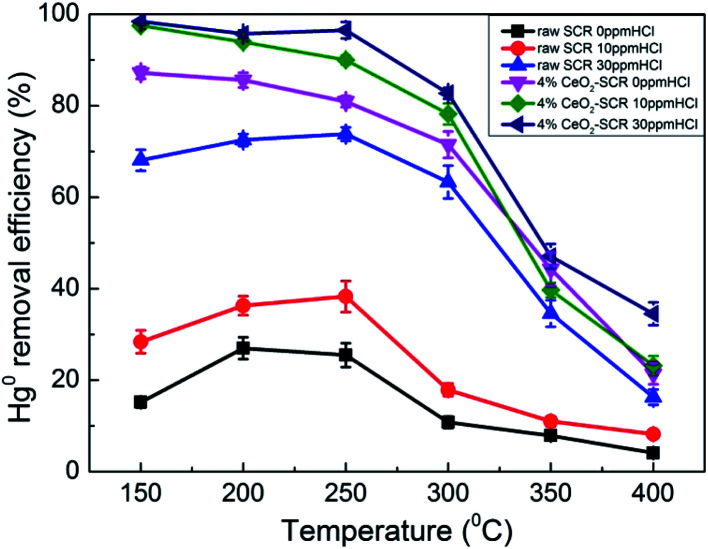
Effect of HCl on Hg^0^ removal efficiency of raw SCR catalyst and 4% CeO_2_-SCR in simulated coal-fired flue gas (reaction gas: SFG with 0, 10, 30 ppm HCl).

As the important oxidant for Hg^0^ oxidation in coal combustion flue gas, effect of HCl on the Hg^0^ removal efficiency of the catalysts was examined, and the results were shown in Fig. 6. For the raw catalyst, Hg^0^ removal efficiency was disadvantaged in the absence of HCl, and the highest value was only 27%. Even though 10 ppm HCl was added into the flue gas, the efficiency was still maintained at a low level since it was below 40% in the whole temperature range. Only when the HCl concentration increased from 10 ppm to 30 ppm did the Hg^0^ removal efficiency of the raw catalyst show a significant improvement. It increased by 35.5% and 45.4%, respectively, at 250 °C and 300 °C as the instances. The above results verified the viewpoint in the literatures that the commercial SCR catalyst was qualified to be utilized in the flue gas derived from burning bitumite with high HCl content while not appropriate to work under low HCl concentration caused by using low-rank coals for Hg^0^ removal.^20,23^ By contrast, after CeO_2_ modification, 4% CeO_2_-SCR exhibited much more prominent Hg^0^ removal efficiency than raw SCR catalyst under the same HCl concentration. The performance over 4% CeO_2_-SCR was even better without HCl than that over the raw catalyst in the presence of 30 ppm HCl. A limited increase of the efficiency of 4% CeO_2_-SCR was observed as the HCl concentration raised. Nevertheless, the catalyst displayed satisfactory Hg^0^ removal activity when exposed to 10 ppm HCl. The Hg^0^ removal efficiencies were excellent at 150–300 °C. Therefore, the CeO_2_ modification weakened the dependence of Hg^0^ removal activity of the catalyst on HCl content of the flue gas. This was really good news for power plants combusting sub-bituminous coal and lignite which occupied the majority of all items. The reason for the superior Hg^0^ removal efficiency of 4% CeO_2_-SCR under low HCl concentration was also due to the improved content of O_ad_ on the catalyst surface. More HCl could be converted by the abundant O_ad_ to form active Cl (Cl*) which had strong oxidation, followed by Hg^0^ being oxidized to HgCl_2_ by Cl*.^51,52^ Through this way, the introduced CeO_2_ enhanced the HCl utilization of the catalyst. The involved reactions could be described by eqn (7) and (8). Meanwhile, this was also one of the main reasons for the higher Hg^0^ removal efficiency over 4% CeO_2_-SCR compared to that over raw SCR in the simulated coal-fired flue gas besides the direct oxidation by O_ad_.

7 2HCl + O_ad_ → 2Cl* + H_2_O

8 Hg^0^ + 2Cl* → HgCl_2_

##### 3.3.2.2. Effect of SO_2_

**Fig. 7:**
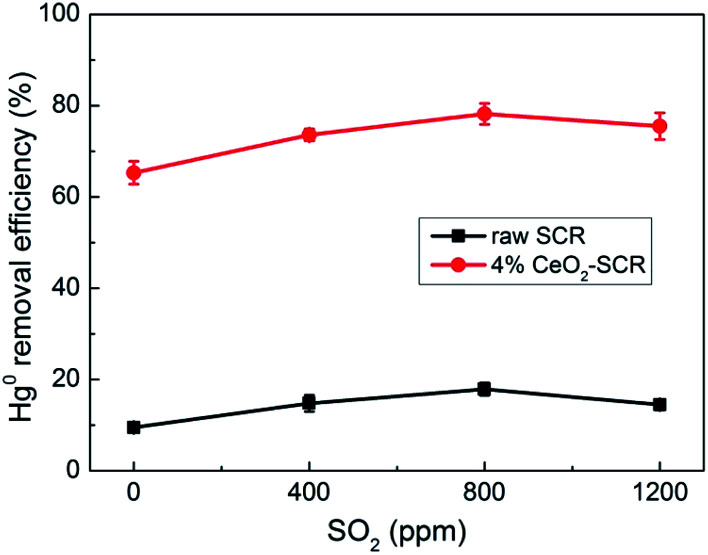
Effect of SO_2_ on Hg^0^ removal efficiency of raw SCR catalyst and 4% CeO_2_-SCR in simulated coal-fired flue gas (reaction gas: SFG with 0, 400, 800, 1200 ppm SO_2_).

Effect of SO_2_ on the Hg^0^ removal efficiency was shown in Fig. 7. The variation trends of Hg^0^ removal efficiency of raw SCR catalyst and 4% CeO_2_-SCR were almost the same with the rising SO_2_ concentration. The efficiency was promoted first as the SO_2_ content in the flue gas increased from 0 to 800 ppm. The promotion could be explained by SO_3_ generated from SO_2_ oxidation, and then Hg^0^ reacted with SO_3_ to form HgSO_4_,^10^ as described by eqn (9) and (10). The increase range of the efficiency was a little larger over 4% CeO_2_-SCR than over raw catalyst, which was probably because the adequate O_ad_ in 4% CeO_2_-SCR converted more SO_2_ to SO_3_ that had the ability to oxidize Hg^0^ and facilitated the proceeding of eqn (10). As SO_2_ content further increased to 1200 ppm, the efficiency suffered slight inhibition, which might be due to the generation of vanadium sulfate and/or cerium sulfate under the high SO_2_ concentration that caused the deactivation of the catalyst to some extent.^53,54^ Compared to the dramatic decrease of the Hg^0^ removal efficiency over Mn-based catalysts in the presence of SO_2_,^34,55^ the commercial V-based catalyst exhibited the advantage of owning excellent sulfur-resistance distinctly.

9 SO_2_ + O_ad_ → SO_3_

10 Hg^0^ + SO_3_ + O_ad_ → HgSO_4_

##### 3.3.2.3. Effects of NO and NH_3_

**Fig. 8:**
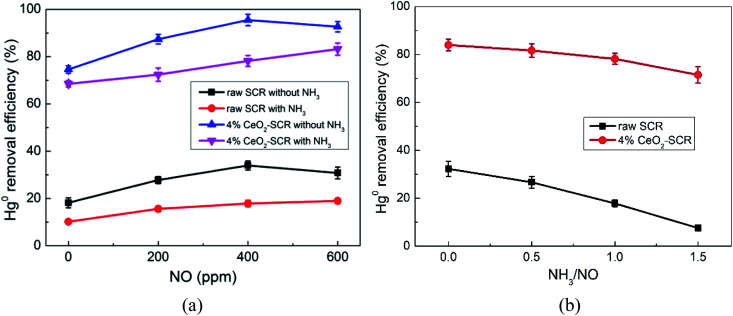
Effects of NO and NH_3_ on Hg^0^ removal efficiency of raw SCR catalyst and 4% CeO_2_-SCR in simulated coal-fired flue gas ((a) effect of NO, reaction gas: SFG with 0, 200, 400, 600 ppm NO in the presence or absence of NH_3_; (b) effect of NH_3_, reaction gas: SFG with 0, 200, 400, 600 ppm NH_3_).

NO and NH_3_ were the principal reactants of the SCR deNO_*x*_ reaction. Effects of NO and NH_3_ in the flue gas on Hg^0^ removal efficiency were important factors for determining the performance of a catalyst for synergistic Hg^0^ removal. The testing results on raw SCR and 4% CeO_2_-SCR were shown in Fig. 8. The increase of NO concentration without the injection of NH_3_ generated the influence of promoting first and then restraining on the efficiencies of both the catalysts, as shown in Fig. 8(a). NO could be oxidized by chemisorbed oxygen on the catalyst to NO_2_ which had the capacity to oxidize Hg^0^ to Hg(NO_3_)_2_.^47^ The related reactions were presented by eqn (11) and (12). And it was the reason for the improvement of the Hg^0^ removal efficiency with the raise of NO concentration. As the NO content further increased after it has reached 400 ppm, the excessive NO would lead to the generation of materials such as nitrite which had no Hg^0^ oxidation capacity and easily caused pore plugging on the catalyst surface besides NO_2_,^56^ resulting in the diminishment of the Hg^0^ removal efficiency. Under the condition of NH_3_ added, the proceeding of SCR deNO_*x*_ reaction removed NO in the flue gas, and the actual concentration of NO was shrunken. Thus, it showed a gradual increase trend of the efficiency as NO content lifted from 0 to 600 ppm, and the inhibition was not formed. Similarly to the effect of SO_2_, the promotion of NO on the efficiency of 4% CeO_2_-SCR was more evident than on the efficiency of raw catalyst, which was owed to the more sufficient O_ad_ in 4% CeO_2_-SCR accelerating the proceeding of eqn (11) and (12) as well. The existence of NH_3_ suppressed Hg^0^ removal efficiency apparently. This judgment could be viewed more intuitively from the results in Fig. 8(b). The increase of the ratio of NH_3_/NO in the flue gas led to obvious inhibitive effect on the efficiencies over both the raw and modified catalysts. NH_3_ was considered to form intense competitive adsorption with Hg^0^ on the surface, hindering the necessary Hg^0^ adsorption process and also the following Hg^0^ oxidation.^42,57,58^ It was worth noting that the inhibition of NH_3_ on the Hg^0^ removal efficiency was weaker over 4% CeO_2_-SCR than over the raw catalyst. The reasonable explanation was that the modified catalyst owned stronger NO removal activity. More NH_3_ was expended in NO removal reaction so that the inhibition on Hg^0^ removal was weakened. In this view, the CeO_2_ modification made the catalyst display better NH_3_-resistance in Hg^0^ removal process, and the property of the catalyst for synergistic Hg^0^ removal was thereby reinforced.

11 NO + O_ad_ → NO_2_

12 Hg^0^ + 2NO_2_ + 2O_ad_ → Hg(NO_3_)_2_

##### 3.3.2.4. Effect of H_2_O

**Fig. 9:**
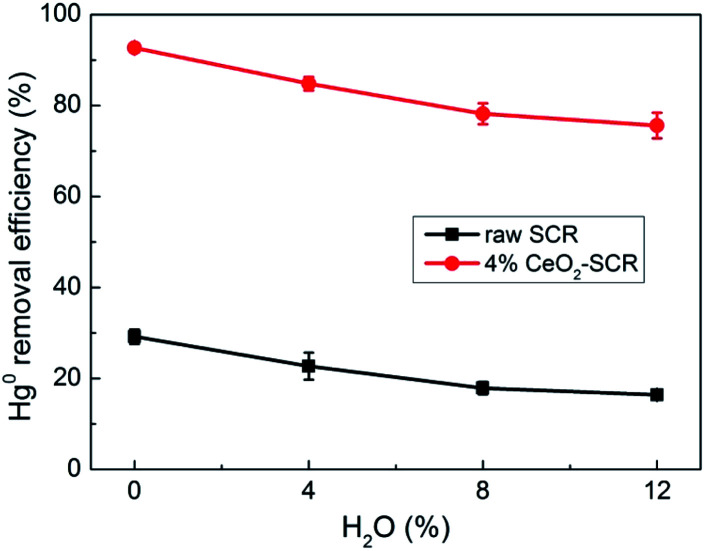
Effect of H_2_O on Hg^0^ removal efficiency of raw SCR catalyst and 4% CeO_2_-SCR in simulated coal-fired flue gas (reaction gas: SFG with 0, 4, 8, 12% H_2_O).

A certain amount of water vapor (H_2_O) was contained in coal-fired flue gas since water was one of the components of coal. Effect of H_2_O on the Hg^0^ removal efficiency was investigated, and the results were shown in Fig. 9. H_2_O generated an unfavorable influence on the efficiency. It declined by a close extent over the raw catalyst and 4% CeO_2_-SCR as 8% H_2_O was added into the flue gas. The inhibitive action could be attributed to the competitive adsorption between H_2_O and the reactants of Hg^0^ oxidation such as Hg^0^ and HCl on the catalyst.^59^ As H_2_O content was augmented from 8% to 12%, the downward trend of the efficiency was visibly diminished, which was perhaps because the common adsorption sites for Hg^0^, HCl and H_2_O were limited and the further increase of H_2_O concentration would not aggravate the inhibition.^34^ Based on the results, the inhibition of H_2_O on the Hg^0^ removal efficiency was not intense in general.

### 3.4. Modification mechanism of CeO_2_ explored by XPS analysis

According to the above experimental results, the CeO_2_ modification generated excellent results on the NO and Hg^0^ removal performance of commercial SCR catalyst. The characterization results of BET and XRD could present the related reasons for the modification effects in a certain degree. In order to further explore the modification mechanism of CeO_2_ on the catalyst, H_2_-TPR and XPS analyses were carried out to detect the redox behavior and valence states (or types) of the elements in the raw and modified catalysts.

#### 3.4.1 H_2_-TPR analysis

**Fig. 10:**
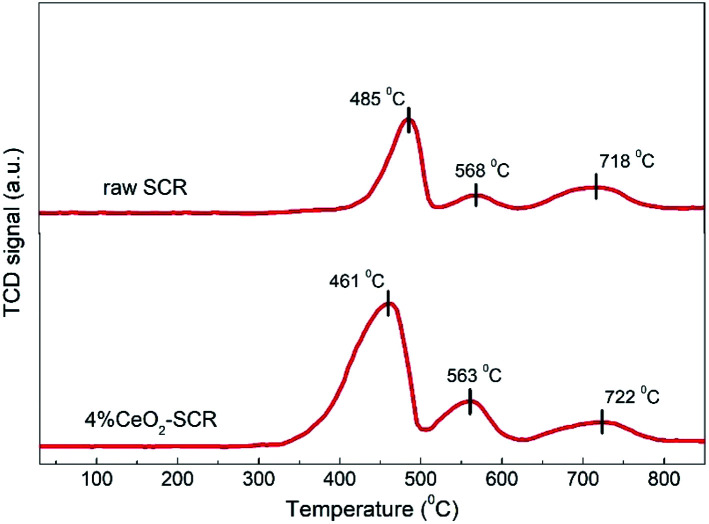
H_2_-TPR profiles of the raw SCR and 4% CeO_2_-SCR catalysts.

H_2_-TPR analysis was implemented over the raw SCR and 4% CeO_2_-SCR catalysts, and the results were shown in Fig. 10. On the profile of the raw catalyst, the peaks emerged at 485 °C and 568 °C could be attributed to the reduction of V^5+^ and surface oxygen, respectively, and the broad shoulder peak at around 720 °C was corresponded to the overlap of the reduction of W^6+^ and lattice oxygen.^44,60,61^ By contrast, a reduction peak was observed at 461 °C on the profile of 4% CeO_2_-SCR. As Ce^4+^ was reported to reduce at about 470 °C, this peak was considered to be the overlapped reduction peak of V^5+^ and Ce^4+^.^62^ It was evident that the temperature of this peak was lowered and the intensity was strengthened dramatically compared to the peak of the raw catalyst at 485 °C, which indicated that the synergy of element V and Ce reinforced the reactivity of the catalyst. In addition, the reduction peak of surface oxygen of 4% CeO_2_-SCR at 563 °C was much stronger than that of the raw catalyst, so it demonstrated the existence of Ce enhanced the oxygen storage capacity of the catalyst. Combining the above factors, the integral area of the reduction profile was obviously larger over 4% CeO_2_-SCR than over the raw catalyst, suggesting the improved redox behavior of the catalyst led by the CeO_2_ modification. The superior redox behavior was favorable to the NO and Hg^0^ removal performance, which was one of main reasons for the prominent catalytic efficiencies of the CeO_2_ modified commercial SCR catalyst.

#### 3.4.2 XPS analysis

**Fig. 11:**
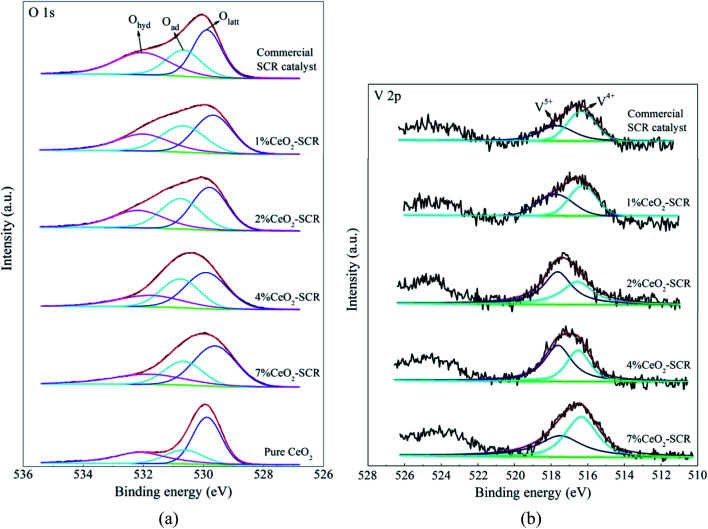
XPS spectra of O 1s and V 2p for the fresh raw and CeO_2_ modified commercial SCR catalysts ((a) O 1s; (b) V 2p).

**Fig. 12:**
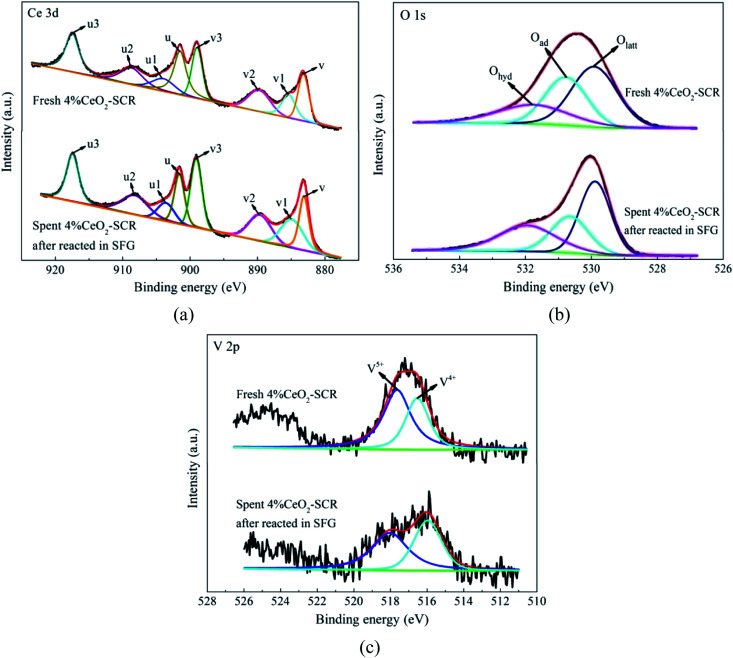
XPS spectra of Ce 3d, O 1s and V 2p for the fresh 4% CeO_2_-SCR catalyst and the spent 4% CeO_2_-SCR catalyst after reacted in SFG ((a) Ce 3d; (b) O 1s; (c) V 2p).

The XPS spectra of the elements for the fresh catalysts, together with the fitting results of the curves, were shown in Fig. 11. For the spectra of O 1s, the fitting peaks were assigned to lattice oxygen (O_latt_), chemisorbed oxygen (O_ad_) and oxygen of hydroxyl and free water (O_hyd_) in sequence at the binding energies from small to large,^25,63^ as shown in Fig. 11(a). And the fitting peaks of V 2p at the binding energies of approximately 516.4 eV and 517.6 eV could be distributed to V^4+^ and V^5+^, respectively,^64,65^ which was shown in Fig. 11(b). In addition, the analysis on the spent catalyst sample of 4% CeO_2_-SCR after reacted in simulated coal-fired flue gas was conducted as well. The obtained curves of Ce 3d, O 1s and V 2p were made comparisons with those of the fresh catalyst, and the results were shown in Fig. 12. On the curves of the element Ce as shown in Fig. 12(a), the fitting peaks of u, u2, u3, v, v2 and v3 were attributed to Ce^4+^, while the peaks of u1 and v1 were corresponded to Ce^3+^.^38,66^ And the spectra of O and V for the spent catalyst were shown respectively in Fig. 12(b) and (c). The ratios of each elemental type or valence state in the corresponding elements of the catalysts were acquired through integrating the fitting peaks and calculating the peak area. The calculation results for the elements of the fresh and spent catalysts were summarized in Tables 3 and 4, respectively.

**Table 3:** The surface atomic contents of O and the ratios of O_ad_ and V^5+^ in the corresponding elements on the catalysts determined by XPS

Catalyst	Content of O (%)	O_ad_/(O_latt_ + O_ad_ + O_hyd_) (%)	Content of O_ad_ (%)	V^5+^/(V^4+^ + V^5+^) (%)
Raw SCR	46.0	26.1	12.0	43.5
1% CeO_2_-SCR	64.9	31.0	20.1	48.0
2% CeO_2_-SCR	64.6	31.4	20.3	56.4
4% CeO_2_-SCR	62.6	32.5	20.4	60.8
7% CeO_2_-SCR	57.4	25.8	14.8	46.1
Pure CeO_2_	46.2	19.8	9.1	—

**Table 4:** The ratios of Ce^3+^, O_ad_ and V^5+^ in the corresponding elements on the fresh and spent 4% CeO_2_-SCR catalysts determined by XPS

Catalyst	Ce^3+^/(Ce^4+^ + Ce^3+^) (%)	O_ad_/(O_latt_ + O_ad_ + O_hyd_) (%)	O_hyd_/(O_latt_ + O_ad_ + O_hyd_) (%)	V^5+^/(V^4+^ + V^5+^) (%)
Fresh 4% CeO_2_-SCR	16.6	32.5	22.8	60.8
Spent 4% CeO_2_-SCR after reacted in SFG	20.5	27.0	30.2	54.7

According to the testing results, the addition of CeO_2_ into the catalyst improved both the surface atomic content of O and the proportion of O_ad_, which led to the increase of the content of O_ad_ on the catalyst, as the data listed in Table 3. It could be judged from the results of Ce 3d of 4% CeO_2_-SCR shown in Fig. 12(a) and Table 4 that Ce^3+^ and Ce^4+^ coexisted in the modified catalysts. The presence of Ce^3+^ with a proportion of 16.6% could create charge imbalance and unsaturated chemical bonds on the surface, which was favorable for the generation of chemisorbed oxygen, thereby raising the content of O_ad_ and boosting the oxygen storage capacity of the catalyst.^38,61,67^ O_ad_ was the active oxygen species that could participate in the catalytic reactions. 4% CeO_2_-SCR owned the highest content of O_ad_ among the catalysts, which was another important reason for its optimal NO and Hg^0^ removal performance. As the CeO_2_ loading increased from 4% to 7%, the O_ad_ content on the catalyst declined and it was even lower than that of the raw catalyst. This result could be associated with the conversion of CeO_2_ to the crystalline phase in 7% CeO_2_-SCR according to the XRD results, which made it disadvantaged for the forming of O_ad_ from the loaded CeO_2_, and meanwhile the forming of crystalline CeO_2_ might consume a number of the intrinsic O_ad_ on the surface. Besides O_ad_, the intensity of the V^5+^ peak and the ratio of V^5+^ were also enlarged with the introduction of CeO_2_. The increase of the V^5+^ proportion might be attributed to part of V^4+^ being oxidized by the abundant O_ad_ to V^5+^ on the modified catalysts. V^5+^ was the active species in V-based catalyst as well, which had good oxidation and was beneficial to Hg^0^ removal activity. So the adequate O_ad_ was also presented in the form of V_2_O_5_. As the content of O_ad_ on the surface of pure CeO_2_ did not show an advantage, it further demonstrated the superior oxygen storage capacity was the result of the synergy of CeO_2_ and V_2_O_5_ in the CeO_2_-SCR catalysts.

After the 4% CeO_2_-SCR catalyst was reacted in SFG, the XPS spectra of O 1s and V 2p for the spent catalyst were compared with those for the fresh one. The results indicated that the intensity of both the O_ad_ and V^5+^ peaks reduced apparently after the reaction, as shown in Fig. 12(b) and (c). The variation could be observed more intuitively by the results in Table 4 that the ratios of O_ad_ and V^5+^ in the corresponding elements decreased from 32.5% to 27% and from 60.8% to 54.7%, respectively, in the reaction process, while the ratio of Ce^3+^ increased from 16.6% to 20.5%. The variation trends of the ratios of O_ad_ and Ce^3+^ on the catalyst were in accordance with those in the related literatures after the catalysts were spent.^19,68^ The decline of the ratio of O_ad_ demonstrated it indeed participated in the catalytic reactions as the active substance. And the decrease of the proportion of V^5+^ suggested the redox behavior between element V and Ce on the catalyst during the reactions, which could be expressed by eqn (13). Combining the eqn (13) with the previous eqn (5), it could be seen that it occurred the process of the redox transformation between Ce^3+^ and Ce^4+^ on the surface, in which the chemisorbed oxygen was generated. The formed O_ad_ then involved in the catalytic reactions such as eqn (6), (7) and (9)–(12) so that the performance of the catalyst for synergistic Hg^0^ removal in SFG was improved. Besides O_ad_, the ratio of O_hyd_ increased by 7.4% in the spent catalyst. On one hand, H_2_O contained in the flue gas adsorbed on the catalyst and formed hydroxyl during the reaction, which caused the competitive adsorption with Hg^0^ and led to the inhibition of H_2_O on Hg^0^ removal efficiency; on the other hand, the increased proportion of O_hyd_ might also be due to the generated H_2_O of eqn (7), thereby further demonstrating the occurrence of this reaction.

13 V^5+^ + Ce^3+^ → V^4+^ + Ce^4+^

**Fig. 13:**
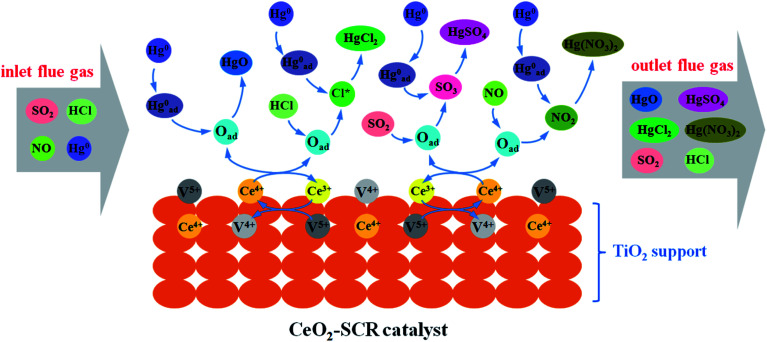
Description of the mechanism of CeO_2_ modification on the synergistic Hg^0^ removal performance of commercial SCR catalyst.

Combining the experimental results and the XPS analysis conclusions, the modification effects of CeO_2_ on commercial SCR catalyst was mainly owed to the more sufficient chemisorbed oxygen which derived from the interaction between element V and Ce and the redox transformation between Ce^3+^ and Ce^4+^ on the catalyst surface. The abundant O_ad_ improved the catalytic activity of the catalyst and the promotion of related flue gas components such as HCl on the Hg^0^ removal efficiency. Integrating these factors, the catalytic property for synergistic Hg^0^ removal was enhanced by the CeO_2_ modification. The modification process was described more vividly and specifically by the illustration shown in Fig. 13.

## 4. Conclusions

CeO_2_ modified commercial SCR catalyst was prepared and investigated for NO and synergistic Hg^0^ removal. The research results indicated that the CeO_2_ loading improved a series of properties of the catalyst. Concretely, the BET surface area, the dispersity of the metal oxides on TiO_2_ support and the redox behavior were increased with the introduction of CeO_2_ into the catalyst, which was favorable to the catalytic activity. The catalyst with the CeO_2_ content of 4% exhibited the optimal performance for simultaneous NO and Hg^0^ removal. The NO conversion of 4% CeO_2_-SCR was as high as 90.5% at 300 °C in SFG with excellent N_2_ selectivity, while the synergistic Hg^0^ removal efficiency could reach 78.2% under the same condition. Owing to the abundant chemisorbed oxygen generated from the synergy of V_2_O_5_ and CeO_2_ and the redox transformation between Ce^3+^ and Ce^4+^, the Hg^0^ removal activity, the HCl utilization and NH_3_-resistance in Hg^0^ removal process and the promotion of SO_2_ and NO on Hg^0^ removal efficiency were improved over 4% CeO_2_-SCR compared to over the raw catalyst. On account of these factors, the CeO_2_ modification made an enhancement on the synergistic Hg^0^ removal performance of the commercial SCR catalyst in simulated coal-fired flue gas, especially under low HCl concentration. Therefore, the CeO_2_ modified commercial SCR catalyst was a potential candidate to be practically applied in coal combustion power plant for NO and synergistic mercury removal.

## Conflicts of interest

There are no conflicts to declare.

## Supplementary Material
